# Doxycycline, metronidazole and isotretinoin: Do they modify microRNA/mRNA expression profiles and function in murine T-cells?

**DOI:** 10.1038/srep37082

**Published:** 2016-11-17

**Authors:** Eugenia Becker, Susan Bengs, Sirisha Aluri, Lennart Opitz, Kirstin Atrott, Claudia Stanzel, Pedro A. Ruiz Castro, Gerhard Rogler, Isabelle Frey-Wagner

**Affiliations:** 1 Division of Gastroenterology and Hepatology, University Hospital Zurich, Zurich, Switzerland; 2 Functional Genomics Center Zurich, Zurich, Switzerland

## Abstract

Inflammatory bowel disease (IBD) may develop due to an inflammatory response to commensal gut microbiota triggered by environmental factors in a genetically susceptible host. Isotretinoin (acne therapy) has been inconsistently associated with IBD onset and flares but prior treatment with antibiotics, also associated with IBD development, complicates the confirmation of this association. Here we studied in mice whether doxycycline, metronidazole or isotretinoin induce epigenetic modifications, and consequently change T-cell mRNA expression and/or function directly after treatment and after a 4 week recovery period. Isotretinoin induced IL-10 signaling in Tregs and naive T-cells directly after treatment and reduced effector T-cell proliferation alone and in co-culture with Tregs. Metronidazole activated processes associated with anti-inflammatory pathways in both T-cell subsets directly after the treatment period whereas doxycycline induced an immediate pro-inflammatory expression profile that resolved after the recovery period. Long-term changes indicated an inhibition of proliferation by doxycycline and induction of beneficial immune and metabolic pathways by metronidazole. Persistent alterations in microRNA and mRNA expression profiles after the recovery period indicate that all three medications may induce long-term epigenetic modifications in both T-cell subsets. Yet, our data do not support the induction of a long-term pro-inflammatory phenotype in murine Tregs and naive T-cells.

Inflammatory bowel diseases (IBD) with the two main forms, Crohn’s disease (CD) and ulcerative colitis (UC), are chronic and relapsing inflammatory conditions of the gastrointestinal tract that affect an increasing number of patients worldwide^1^. The precise pathogenesis of IBD is multifactorial and is not completely elucidated yet. It is generally considered that environmental factors represent an important contributor to the pathogenesis of IBD by triggering an inappropriate and progressive immune response to the commensal gut microbiota in a genetically predisposed host^2^. To date, genome-wide association studies have identified more than 240 IBD susceptibility loci, affecting genes involved in immune regulation, mucosal immune response, autophagy and epithelial barrier function^3^,^4^,^5^.

Currently available data support the concept that IBD is a polygenic disease and suggest that non-genetic modifications involved in regulatory processes might have an impact on susceptibility and severity of disease^6^. Mechanisms of gene regulation that do not alter the basic sequence of DNA are called epigenetic regulations and have been studied extensively over the last few years. These studies have shown that epigenetic modifications are associated with a variety of diseases, e.g. IBD^7^,^8^, multiple sclerosis^9^, psoriasis^10^ and systemic lupus erythematosus^11^. The molecular basis of epigenetic regulation is complex. Importantly, changes may remain through cell division, and last for many generations (long-term effects)^12^. So far, three major epigenetic modifications have been identified: DNA methylation, histone modifications and differential microRNA expression. MicroRNAs are small non-coding RNA (18–24 nucleotides) molecules binding to complementary sequences in the 3′ untranslated region (3′-UTR) of their target mRNAs leading to sequestration, degradation or storage of these target mRNAs^13^.

Mounting evidence demonstrates that antibiotics can impact on the occurrence of IBD and IBD flares, as well as increase the risk of developing IBD in children and adults, indicating long-term effects after medication with antibiotics^14^,^15^,^16^,^17^,^18^. Moreover, some case reports have shown a potential association between isotretinoin, a non-antimicrobial drug used for the treatment of severe acne, and the development of IBD during medication, immediately after or even weeks or months after cessation of therapy^19^,^20^,^21^. Yet, others were unable to confirm these findings^22^,^23^,^24^. Isotretinoin is typically used in patients unresponsive to antibiotics^15^,^25^. Hence, any attempt to confirm a causal relationship between isotretinoin and IBD is confounded by prior antibiotic treatment.

In the present study, we have determined microRNA expression, mRNA expression as well as functional parameters in murine naive and regulatory T-cells to evaluate immunomodulatory effects of doxycycline (used in acne therapy and associated with IBD), metronidazole (one of the preferred antibiotics in IBD treatment), and isotretinoin. To capture immediate and persistent effects, samples were taken directly after a 2 week treatment period and 4 weeks after the last drug administration. The latter time point is henceforth referred to as recovery phase.

## Results

**Figure 1:**
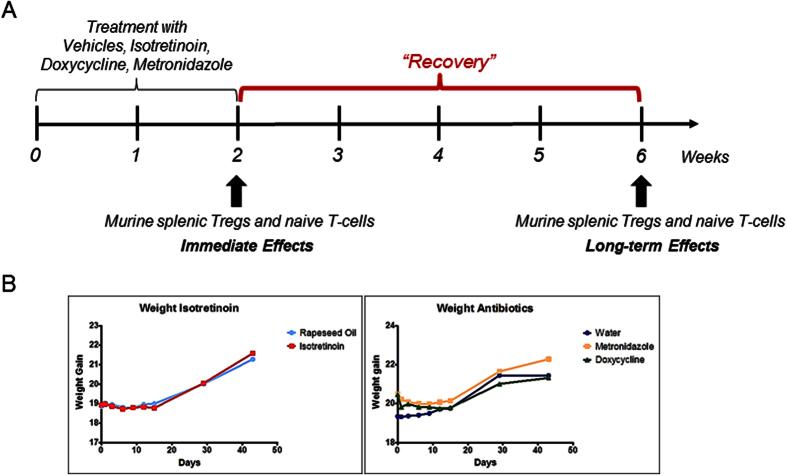
Experimental design. BALB/c female mice were treated with isotretinoin, rapeseed oil (isotretinoin vehicle), metronidazole, doxycycline or water (antibiotics vehicle) by daily oral gavage for 2 weeks. (**A**) Splenic T-cells were isolated after 2 weeks of treatment (immediate effects) and after a recovery phase of 4 weeks after treatment cessation (long-term effects). For isotretinoin and rapeseed oil, 2-5 replicates (pool of 2-4 animals or no pooling) per group were sampled immediately after treatment and 2-5 replicates after the recovery phase. For metronidazole, doxycycline and water, 2 replicates (pool of 6 animals) were sampled per time point and group. (**B**) No differences between treatment groups were registered with respect to body weight for all time points.

In order to investigate direct and long-term alterations in murine splenic T-cell subsets upon oral isotretinoin, metronidazole or doxycycline treatment (Fig. 1), microRNA and mRNA expression were assessed by Next-Generation Sequencing. We first analyzed direct and long-term effects in naive T-cells (CD4+CD62L+) to study an influence on largely undifferentiated cells. We further investigated the impact on Tregs (CD4+CD25+) to unravel modifications that might impair Tregs in a way that favors an inflammatory status.

### Correlation of predicted microRNA targets and mRNA expression directly after isotretinoin or antibiotic treatment in naive T-cells

**Figure 2:**
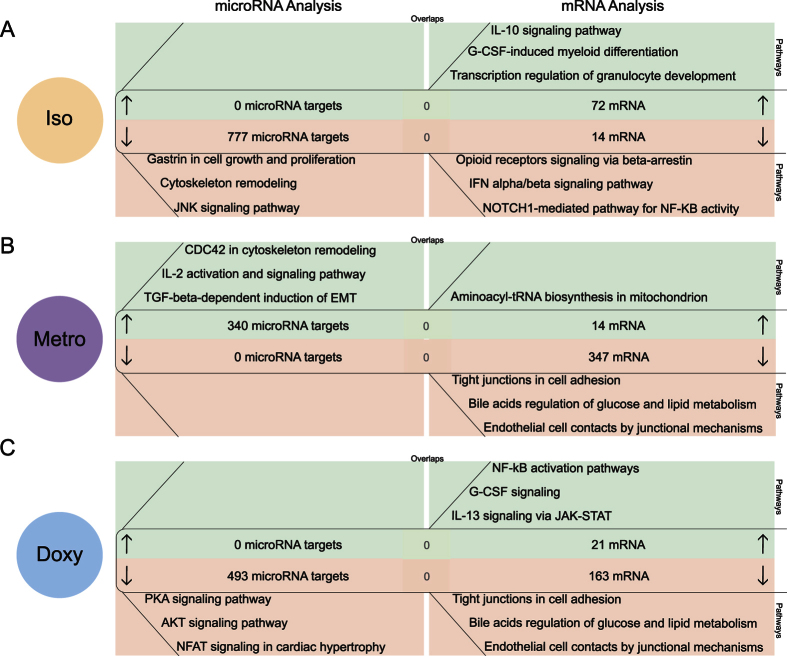
Correlation of predicted microRNA targets and mRNA expression directly after isotretinoin or antibiotic treatment in naive T-cells (direct effect). Analysis of mathematically predicted microRNA targets was performed with TargetScan or TargetScan custom for (**A**) isotretinoin (iso), (**B**) metronidazole (metro) and (**C**) doxycycline (doxy) treated animals compared to control groups (left panels). Identification of differentially expressed mRNAs was performed with the following thresholds: |log2 (fold change)| ≥0.5, P-value ≤ 0.001 (right panels). Three of the most significant pathways associated with potentially up-regulated microRNA targets as predicted from down-regulated microRNAs and mRNAs are shown in the green sections whereas down-regulated microRNA targets (as predicted from up-regulated microRNAs) and mRNAs are depicted in the red sections. Shared candidates between predicted microRNA targets and experimentally derived mRNAs are displayed in the centers of the respective section. The pathway analysis was performed with MetaCore by Thomson Reuters.

All three orally administered agents led to differentially expressed microRNAs, as defined by (|log2 (fold change)| ≥1, P-value ≤ 0.001), in naive T-cells directly after the treatment period (Fig. 2). 3 microRNAs (microRNA-3971, −3962, −374c-5p) were significantly overexpressed in isotretinoin treated animals and potentially down-regulate 777 microRNA targets as defined with TargetScan (Fig. 2A). Pathway analysis by MetaCore of these microRNA targets identified gastrin-mediated effects on cell growth and proliferation (P = 8.47E-08), cytoskeleton remodeling (P = 9.60E-08) and the c-Jun N terminal kinase (JNK) signaling pathway (P = 1.31E-07) as the most strongly affected. Interestingly, the transcriptome analysis of naive T-cells revealed that 2 weeks of isotretinoin treatment led to an increase in the expression of mRNAs, as defined by (|log2 (fold change)| ≥0.5, P-value ≤ 0.001), involved in interleukin (IL)-10 signaling (*Bcl-2, Fc gamma RIIalpha, Mmp-9* and *Socs3*) (P = 3.64E-07) and a down-regulation of genes involved in pro-inflammatory processes, such as interferon (IFN) signaling (*Isg15*) (P = 1.30E-02) and nuclear factor (NF)κB activity (*Histone 4*) (P = 1.84E-02) suggesting a direct anti-inflammatory effect of isotretinoin on naive T-cells. No overlap between predicted microRNA targets and differentially expressed mRNAs could be observed.

Upon metronidazole treatment, the expression of 5 microRNAs was significantly lower as compared to the respective control group (Supplementary Table 1). The down-regulation of these microRNAs resulted in the prediction of 340 potentially up-regulated microRNA targets associated with IL-2 activation and signaling (P = 3.41E-06), cytoskeleton remodeling (P = 8.53E-06) and epithelial-to-mesenchymal-transition (EMT) (P = 2.20E-06) (Fig. 2B). However, metronidazole treatment had only minor effects on mRNA expression in splenic naive T-cells, with only 14 mRNAs being significantly overexpressed in comparison to the control group. Among these mRNAs, IL-17r was strongly up-regulated (14-fold, P  =  1.80E-06) directly after treatment. However, Metacorewas unable to build a network around IL-17r despite its strong up-regulation (Fig. 2B). There was no overlap between predicted microRNA targets and differentially expressed mRNAs.

Doxycycline treatment induced significant overexpression of microRNA-144-3p that resulted in the prediction of 493 potentially down-regulated microRNA targets involved in protein kinase A (PKA) (P = 9.01E-06), protein kinase B (known as AKT) (P = 2.42E-04) and nuclear factor of activated T-cells (NFAT) signaling pathways (P = 3.81E-04) (Fig. 2C and Supplementary Table 1). Transcriptome analysis of doxycycline treated animals showed an up-regulation of 21 mRNAs involved in pro-inflammatory pathways, such as NFκB signaling (*Baff, IL-1beta*) (P = 3.42E-03) and IL-13 signaling (*Alox15, Rsnb*) (P = 2.56E-03). Notably, metronidazole and doxycycline strongly downregulated mRNAs involved in cell adhesion (P = 1.41E-06; P = 5.15E-08) and metabolism processes (P = 2.47E-05; 2.15E-06) in naive T-cells directly after treatment (Fig. 2B and C, right). No overlap between predicted microRNA targets and differentially expressed mRNAs could be observed.

### Correlation of predicted microRNA targets and mRNA expression after the recovery phase in naive T-cells of isotretinoin and antibiotic treated animals

**Figure 3:**
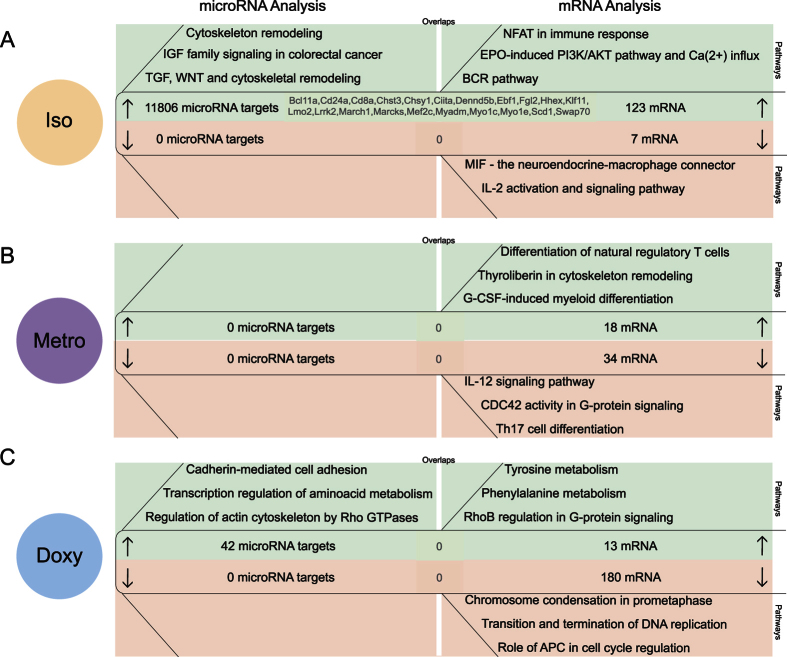
Correlation of predicted microRNA targets and mRNA expression after the recovery phase in naive T-cells of isotretinoin and antibiotic treated animals (long-term effect). Analysis of mathematically predicted microRNA targets was performed with TargetScan or TargetScan custom for (**A**) isotretinoin (iso), (**B**) metronidazole (metro) and (**C**) doxycycline (doxy) treated animals compared to control groups (left panels). Differentially expressed mRNAs were identified with the following thresholds: |log2 (fold change)| ≥0.5, P-value ≤ 0.001 (right panels). Three of the most significant pathways associated with up-regulated microRNA targets (down-regulated microRNAs) and mRNAs are shown in the green sections whereas down-regulated microRNA targets (up-regulated microRNAs) and mRNAs are depicted in the red sections. Shared candidates between predicted microRNA targets and experimentally derived mRNAs are displayed in the centers of the respective section. The pathway analysis was performed with MetaCore by Thomson Reuters.

After the 4-week recovery period (Fig. 3), previous treatment with isotretinoin was followed by a down-regulation of 35 microRNAs in naive T-cells. This large number of differentially expressed microRNAs led to a prediction of 11′806 potentially up-regulated microRNA targets predominantly involved in cytoskeleton remodeling (P = 1.70E-18) and insulin-growth-factor signaling (P = 9.61E-17). When we analyzed changes in mRNA expression after the recovery period in isotretinoin treated animals, significantly overexpressed mRNAs were involved in Ca(^2+^)- and NFAT signaling (P = 1.70E-05; 3.96E-05) as well as in B cell receptor-associated pathways (P = 5.89E-09). We found 21 overlaps between mathematically derived microRNA targets and experimentally derived mRNAs. The mRNAs thereof are involved in signal transduction in immune cells, e.g. *Ciita, Lrrk2, Marcks*, and antigen presentation responses (*CD24a, CD8a*), suggesting relevant epigenetic changes important for T-cell function. Isotretinoin led to a significant down-regulation of 7 mRNAs only, indicating minor effects on mRNA suppression in naive T-cells after the recovery phase.

Metronidazole treatment had no significant long-term effects on microRNA expression in naive T-cells in comparison to the control group. Despite a lack of epigenetic regulation at microRNA level, metronidazole treatment led to increased expression of a number of mRNAs after the recovery period. These were involved in cytoskeleton remodeling (P = 2.13E-02) and, surprisingly, in the differentiation into Tregs (P = 2.26E-02) through up-regulation of *Foxp3* (Supplementary Table 2). However, the number of mRNAs with significantly higher expression was relatively small (18 mRNAs) indicating a moderate long-term effect. In the opposite direction, metronidazole treatment led to a reduced expression of 34 mRNAs which were identified to be associated with IL-12 signaling (P = 1.49E-02) and T helper (Th)17 differentiation (*Irf4*) (P = 2.26E-02). The up-regulation of the expression of Treg differentiation factors and the down-regulation of expression of Th1 and Th17 associated mRNAs may be interpreted as an overall anti-inflammatory long-term effect.

Doxycycline treatment significantly decreased the expression of only 1 microRNA (microRNA-144-5p) after the recovery period in naive T-cells. The decrease of microRNA-144-5p resulted in the prediction of 42 potentially up-regulated microRNA targets involved in cell adhesion (P = 1.12E-03), cytoskeleton (P = 8.73E-04) and metabolism (P = 1.03E-03) pathways. At transcriptome level 13 mRNAs likewise involved in metabolism (P = 1.40E-02) and G-protein signaling (P = 3.47E-03) were overexpressed, indicating consistent regulation at microRNA and mRNA level. However, no overlaps between predicted microRNA targets and differentially expressed mRNAs were determined indicating a minor effect of microRNA mediated regulation on gene expression in naive T-cells after the recovery phase in doxycycline treated animals.

### Correlation of predicted microRNA targets and mRNA expression directly after isotretinoin and antibiotic treatment in Tregs

In addition to the analysis of naive T-cells, we analyzed changes in the expression of microRNAs in Tregs directly after the treatment (immediate effects).

**Figure 4:**
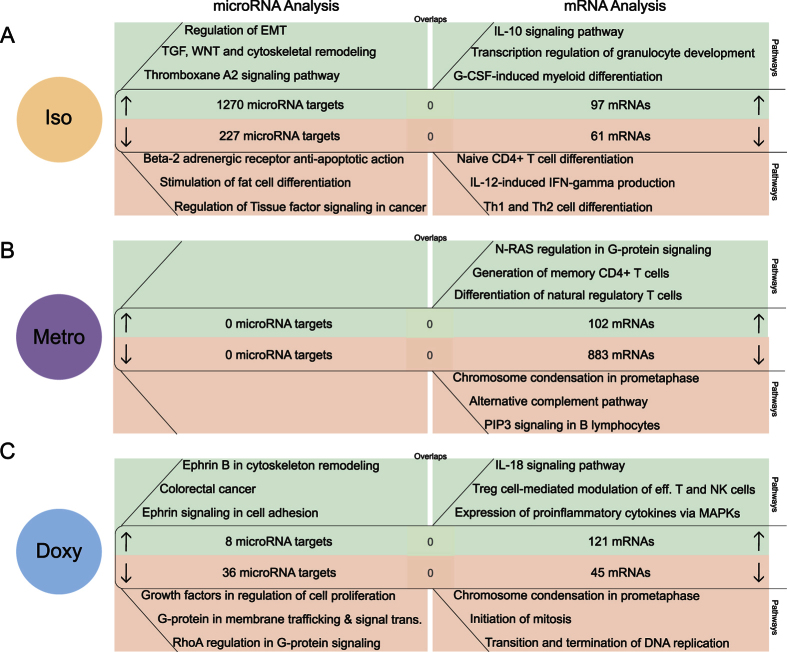
Correlation of predicted microRNA targets and mRNA expression directly after isotretinoin or antibiotic treatment in Tregs (direct effect). Analysis of mathematically predicted microRNA targets was performed with TargetScan or TargetScan custom for (**A**) isotretinoin (iso), (**B**) metronidazole (metro) and (**C**) doxycycline (doxy) treated animals compared to control groups (left panels). Differentially expressed mRNAs were identified with the following thresholds: (|log2 (fold change)| ≥0.5, P-value ≤ 0.001 (right panels). Three of the most significant pathways associated with up-regulated microRNA targets (down-regulated microRNAs) and mRNAs are shown in the green sections whereas down-regulated microRNA targets (up-regulated microRNAs) and mRNAs are depicted in the red sections. Shared candidates between predicted microRNA targets and experimentally derived mRNAs are displayed in the centers of the respective section. The pathway analysis was performed with MetaCore by Thomson Reuters.

Treatment with isotretinoin induced overexpression of 7 microRNAs, which potentially down-regulate 227 microRNA targets involved in apoptosis (P = 7.44E-06), fat cell differentiation (P = 2.91E-05) and cancer pathways (P = 9.54E-05) (Fig. 4A). For the 4 down-regulated microRNAs after isotretinoin treatment, 1′270 potentially up-regulated mRNAs were predicted by TargetScan being involved in cytoskeleton remodeling (P = 5.42E-09) and developmental pathways (P = 2.14E-08). Interestingly, mRNA expression analysis showed that IL-10 signaling (*CD23, Ikbz, Mmp-9* and *Socs3*) (P = 4.20E-07) was activated upon isotretinoin treatment also in Tregs, like we had found for naive T-cells, indicating a direct anti-inflammatory effect of isotretinoin on both T-cell subsets. Significant down-regulation of mRNAs involved in IL-12 signaling (P = 2.53E-04) and Th1/Th2 differentiation (*Ifnγ, IL-18r1, T-bet, CD40L*) (P = 3.47E-04) in Tregs directly after isotretinoin treatment further supports a direct anti-inflammatory effect of isotretinoin. There was no overlap between predicted microRNA targets and differentially expressed mRNAs.

Directly after metronidazole treatment microRNA expression was not affected. Pathway analysis of the 102 overexpressed mRNAs in response to metronidazole treatment revealed that activation of genes involved in G-protein signaling (P = 4.87E-04), memory generation (P = 2.56E-05) and - like for the long-term effect in naive T-cells - differentiation of natural Tregs (*CD4, Foxp3, Gitr, CD25, TCRalpha/beta*) (P = 5.46E-07). In the opposite direction, 883 mRNAs involved in cell cycle pathways (P = 2.08E-12) and immune responses (P = 7.56E-11) were significantly down-regulated (Fig. 4B).

Doxycycline treatment had a direct impact on the expression of 2 microRNAs in Tregs: microRNA-100-5p and -1249-3p. The up-regulation of microRNA-100-5p predicts the down-regulation of 36 microRNA targets linked to G-protein-associated signaling (P = 4.08E-04) and cell proliferation processes (P = 1.53E-04). The 8 mRNAs that could be targeted by microRNA-1249-3p are important in Ephrin signaling (P = 2.265E-4) and colorectal cancer (P = 1.602e-2). Doxycycline increased the expression of 121 mRNAs associated with pro-inflammatory responses, such as IL-18 signaling (*Cox-2, IL-1β, IL18rap, Mmp-9*) (P = 3.11E-04), and down-regulated the expression of 45 mRNAs involved in cell cycle processes (P = 1.81E-09). However, none of these mRNAs were among the mathematically predicted microRNA targets. In summary, doxycycline appears to induce inflammatory processes while inhibiting proliferation in Tregs immediately after treatment, yet no epigenetic regulation by microRNAs seems to be responsible for the effects observed at mRNA level.

### Correlation of predicted microRNA targets and mRNA expression after the recovery period in Tregs of isotretinoin and antibiotic treated animals

**Figure 5:**
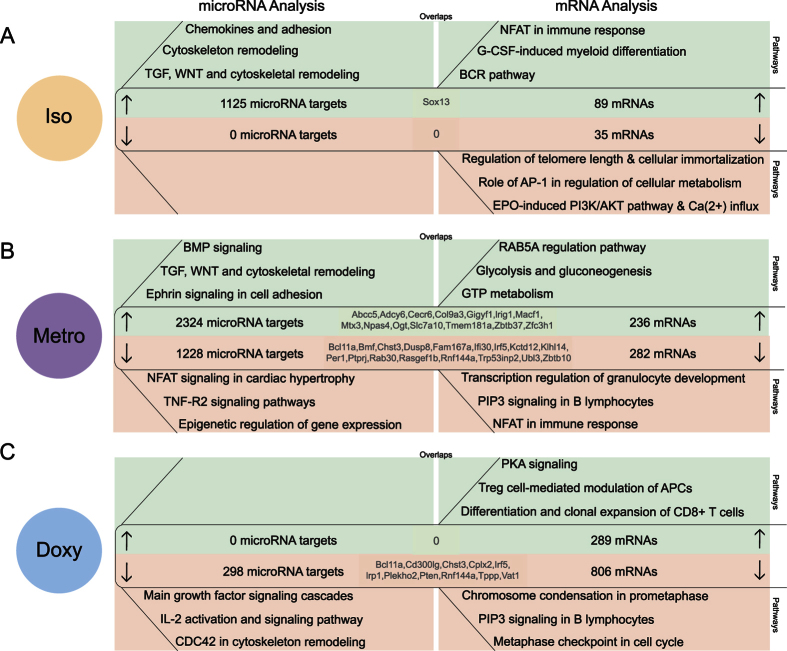
Correlation of predicted microRNA targets and mRNA expression after the recovery phase in Tregs of isotretinoin and antibiotic treated animals (long-term effect). Analysis of mathematically predicted microRNA targets was performed with TargetScan or TargetScan custom for (**A**) isotretinoin (iso), (**B**) metronidazole (metro) and (**C**) doxycycline (doxy) treated animals compared to control groups (left panels). Selection of differentially expressed mRNAs was performed with the following thresholds: |log2 (fold change)| ≥0.5, P-value ≤ 0.001 (right panels). Three of the most significant pathways associated with up-regulated microRNA targets (down-regulated microRNAs) and mRNAs are shown in the green sections whereas down-regulated microRNA targets (up-regulated microRNAs) and mRNAs are depicted in the red sections. Shared candidates between predicted microRNA targets and experimentally derived mRNAs are displayed in the centers of the respective section. The pathway analysis was performed with MetaCore by Thomson Reuters.

After the recovery phase, significant changes in microRNA expression could be observed in Tregs of animals treated with isotretinoin, metronidazole or doxycycline treated animals, with overlaps between predicted microRNA target and mRNA data sets within the respective treatment groups indicating possible epigenetic regulation (Fig. 5).

Previous isotretinoin treatment led to significantly reduced expression of 5 microRNAs resulting in the mathematical prediction of 1′125 potentially up-regulated mRNAs by TargetScan. These predicted microRNA targets were annotated to be involved in cell adhesion (P = 3.96E-07) and cytoskeleton remodeling processes (P = 7.98E-12). When correlating the prediction based microRNA data with overexpressed mRNAs, we identified 1 overlap, *Sox13* (Fig. 5A). The transcriptome analysis further showed a significant overexpression of mRNAs implicated in NFAT signaling (P = 5.19E-08), granulocyte-colony stimulating factor (G-CSF) induced differentiation (P = 6.29E-06) and B-cell receptor (BCR) responses (P = 5.91E-13), whereas down-regulated mRNAs were associated with cell cycle regulation (P = 1.89E-02), as well as activator protein (AP)-1 (P = 2.05E-02) and Ca(^2+^)-dependent processes (P = 2.32E-02).

After the recovery period, previous metronidazole treatment affected the expression of a total of 11 microRNAs in Tregs. For the 8 microRNAs with significantly increased expression, 1′228 possible microRNA targets were predicted and were enriched in different pathways, such as NFAT (P = 7.01E-10) and tumor necrosis factor receptor (TNFR)2 signaling (P = 1.98E-08), as well as in epigenetic regulation of gene expression (P = 4.21E-07). Correlating these potentially down-regulated microRNA targets with the 282 down-regulated mRNAs, we found 17 common mRNAs between both data sets. The 282 down-regulated mRNAs were involved in transcriptional regulation, phosphatidylinositol 3,4,5-trisphosphate (PIP3) (P = 4.13E-06) and NFAT signaling (P = 1.57E-05) (Fig. 5B). Moreover, the down-regulation of 3 microRNAs (microRNA-30e-5p, -340-5p, -142b) resulted in a prediction of 2′324 potentially up-regulated microRNA targets associated with bone morphogenetic proteins (BMP) (P = 5.328E-8), Ephrin (P = 2.44E-12) and cytoskeleton remodeling processes (P = 1.68E-07). In animals treated previously with metronidazole we observed an increase in the expression of 236 mRNAs after the recovery period, known to be involved in metabolism (P = 7.81E-03) and Rab5a-dependent transport pathways (P = 8.46E-03). 14 mRNAs were shared between the up-regulated microRNA target and mRNA data sets suggesting possible epigenetic regulation.

Previous doxycycline treatment led to differential expression of 4 microRNAs in Tregs. The analysis of microRNA targets of the microRNA-486 family indicated 298 potentially down-regulated mRNAs. 11 of those were also found within the data set of significantly downregulated mRNAs (806 mRNAs). The affected processes included IL-2 (P = 1.71E-06), CDC42 signaling (P = 5.16E-06) and cell growth (P = 4.86E-07) for the down-regulated microRNA targets, and cell cycle (P = 2.63E-09) and PIP3 signaling (P = 6.02E-09) for the down-regulated mRNAs. Pathway analysis of the 289 up-regulated mRNAs indicated that the expression of genes involved in immune responses, such as Treg function (*Ctla-4, IL-10*) (P = 5.16E-04), PKA signaling (P = 1.22E-03) and differentiation of CD8+ T-cells (P = 4.37E-04) was affected, suggesting a rather immunosuppressive phenotype in Tregs after the recovery phase in doxycycline treated animals.

### Isotretinoin inhibits effector T-cell proliferation immediately after treatment cessation, but does not provoke long-term effects

To explore the direct and long-term effects of isotretinoin on T-cell function, we assessed the proliferation of effector T-cells (Teffs; CD4+CD25−) by monitoring the fluorescence signal of carboxyfluoresceinsuccinimidyl ester (CFSE). Teffs from vehicle- or isotretinoin-treated animals were co-cultured either with Tregs from mice under the same treatment (e.g. Treg(ctrl)/Teff(ctrl)) or combining different treatments in a mixed setting, e.g. Treg(ctrl)/Teff(iso), to discriminate how T-cell proliferation is affected by isotretinoin treatment.

**Figure 6:**
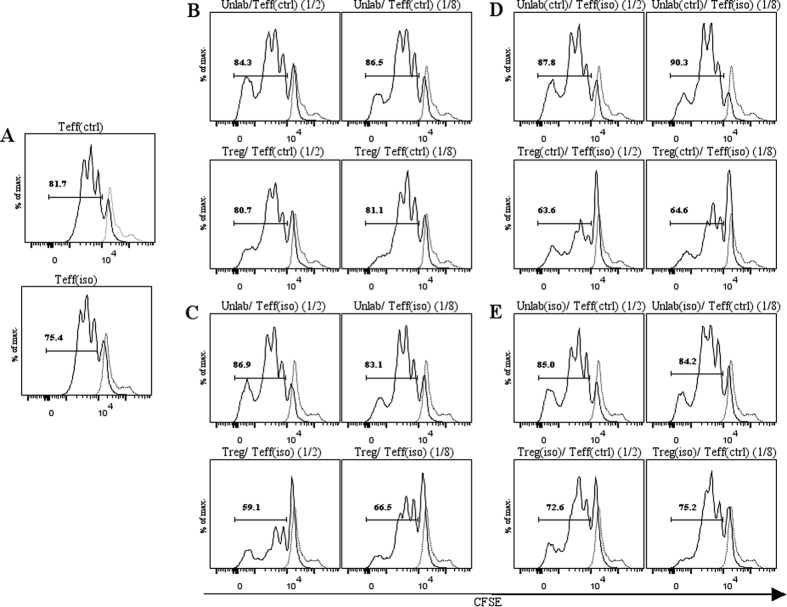
Immediate effects of isotretinoin on Treg suppression/Teff proliferation. Proliferation of Teffs was assessed by FACS analysis by measuring the dilution of the intensity of CFSE. (**A**) Proliferation of Teffs for the control group (top) and isotretinoin group (bottom) at basal conditions. (**B**) Unlabeled Teffs co-cultured with CFSE-labeled Teffs from vehicle treated animals at a 1/2 or 1/8 ratio (top) or Tregs co-cultured with Teffs from the controls at a 1/2 or 1/8 ratio (bottom). (**C**) Unlabeled Teffs co-cultured with CFSE-labeled Teffs from isotretinoin treated animals at a 1/2 or 1/8 ratio (top) or Tregs co-cultured with Teffs from the controls at a 1/2 or 1/8 ratio (bottom). (**D**) Inverse setting: unlabeled Teffs from controls were cultured with CFSE-labeled Teffs from isotretinoin treated animals at a 1/2 or 1/8 ratio or Tregs from controls cultured with Teffs from the isotretinoin condition. (**E**) Inverse setting: unlabeled Teffs from isotretinoin treated animals were cultured with CFSE-labeled Teffs from control animals at a 1/2 or 1/8 ratio or Tregs from isotretinoin treated animals were cultured with Teffs from controls. Shown are representative graphs of two independent experiments. Solid line: stimulated CFSE-labeled Teffs. Dashed line: unstimulated CFSE-labeled Teffs. Abbreviations: Teffs, effector T-cells (CD4+CD25−); Tregs, regulatory T-cells (CD4+CD25+); unlab, unlabeled Teffs; ctrl, vehicle treated animals; iso, isotretinoin treated animals; 1/2 and 1/8 ratio of unlab or Tregs; FACS, fluorescence-activated cell sorting; CFSE, carboxyfluoresceinsuccinimidyl ester.

Interestingly, stimulated Teff(iso) proliferated less compared to Teff(ctrl) under basal conditions (Fig. 6A). Co-cultures of Teff(ctrl) or Teff(iso) with unlabeled Teffs (unlab) at a 1/2 or 1/8 ratio under the same treatment conditions, showed similar proliferation patterns (Fig. 6B and C upper panels). However, when co-culturing Teff(iso) with Treg(iso) at a 1/2 ratio, the inhibition of Teff(iso) proliferation was enhanced compared to Teff(ctrl) co-cultured with Treg(ctrl) (Fig. 6B and C lower panels). These effects were partly reversed when Treg(iso) were cultured at a 1/8 ratio.

Culturing T-cells using different combinations (Teff(ctrl)/Treg(iso) and Teff(iso)/Treg(ctrl)) we observed reduced proliferation of Teff(iso) co-cultured with Treg(ctrl) compared to Teff(ctrl) cultured with Treg(iso) (Fig. 6D and E lower panels) while proliferation in controls (unlab/Teff(iso or ctrl)) was unaffected (Fig. 6D and E upper panels). In summary, these results indicate that Teff isolated from isotretinoin treated animals are more sensitive to inhibition of proliferation by Tregs, independently of whether the Tregs had been exposed to isotretinoin or not.

**Figure 7:**
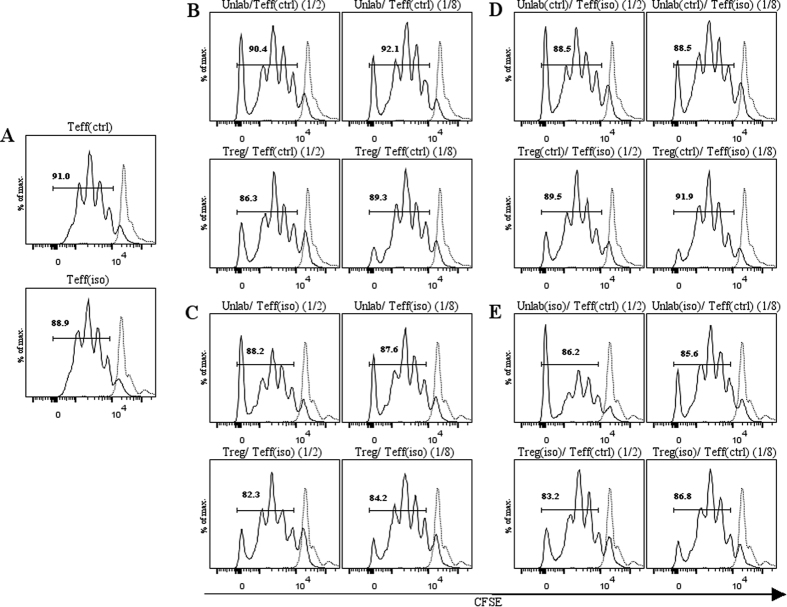
Long-term effects of isotretinoin on Treg suppression/Teff proliferation. Proliferation of Teffs was assessed by FACS analysis by measuring the dilution of the intensity of CFSE. (**A**) Proliferation of Teffs for the control group (top) and isotretinoin group (bottom) at basal conditions. (**B**) Unlabeled Teffs co-cultured with CFSE-labeled Teffs from vehicle treated animals at a 1/2 or 1/8 ratio (top) or Tregs co-cultured with Teffs from the controls at a 1/2 or 1/8 ratio (bottom). (**C**) Unlabeled Teffs co-cultured with CFSE-labeled Teffs from isotretinoin treated animals at a 1/2 or 1/8 ratio (top) or Tregs co-cultured with Teffs from the controls at a 1/2 or 1/8 ratio (bottom). (**D**) Inverse setting: unlabeled Teffs from controls were cultured with CFSE-labeled Teffs from isotretinoin treated animals at a 1/2 or 1/8 ratio or Tregs from controls cultured with Teffs from the isotretinoin condition. Inverse setting: unlabeled Teffs from isotretinoin treated animals were cultured with CFSE-labeled Teffs from control animals at a 1/2 or 1/8 ratio or Tregs from isotretinoin treated animals were cultured with Teffs from controls. Shown are representative graphs of three independent experiments. Solid line: stimulated CFSE-labeled Teffs. Dashed line: unstimulated CFSE-labeled Teffs. Abbreviations: Teffs, effector T-cells (CD4+CD25−); Tregs, regulatory T-cells (CD4+CD25+); Unlab, unlabeled Teffs; ctrl, vehicle treated animals; iso, isotretinoin treated animals; 1/2 and 1/8 dilution of Unlab or Tregs; FACS, fluorescence-activated cell sorting; CFSE, carboxyfluoresceinsuccinimidyl ester.

When we performed the experiment with T-cells isolated after the 4-weeks recovery period to study long-term effects, we did not observe any significant differences in Teff proliferation (Fig. 7).

### Isotretinoin induces Socs3 protein expression in Jurkat cells, but does not activate the p38-ERK-MSK1 signaling pathway

**Figure 8:**
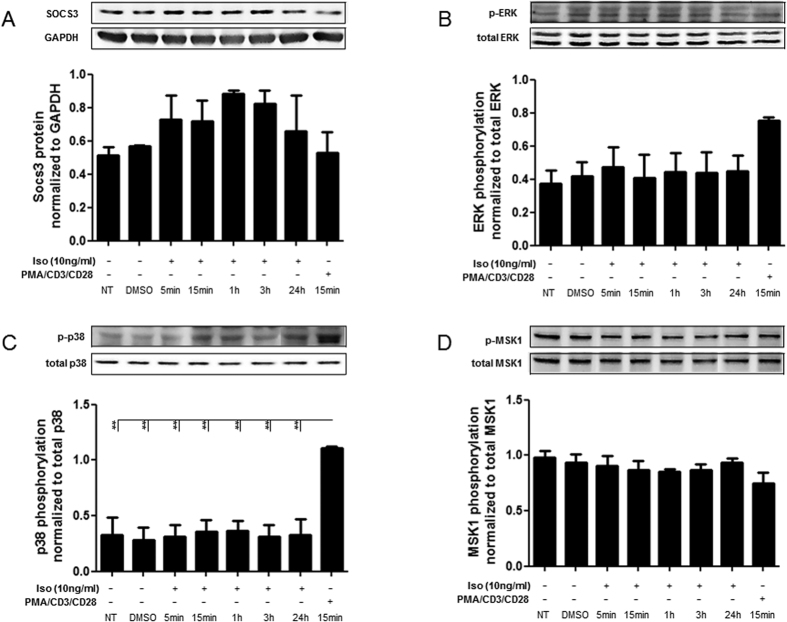
Isotretinoin influences Socs3 expression in Jurkat cells but does not activate the p38-ERK-MSK1 signaling pathway. Jurkat cells were treated with isotretinoin (10 ^ng^/_ml_) and PMA (100 ^ng^/_ml_) in combination with anti-CD3/CD28 stimulation and samples were taken at the indicated time points. (A-D) Representative Western Blots show levels of (**A**) Socs3, (**B**) p-ERK (Tyr^42^/Tyr^44^), (**C**) p-p38 (Thr^180^/Tyr^182^) and (**D**) p-MSK1 (Thr^581^). The blots were cropped for improving clarity and full-length blots are presented in Supplementary Figure 2. The solid line on each blot depicts a vertically sliced area where the gel size maker was cropped. Expression was normalized to total protein content or anti-GAPDH (loading control); the graphs depict densitometric analysis (n = 3). Significant differences in comparison to the controls are denoted by asterisk (*P ≤ 0.05, **P ≤ 0.01, ***P ≤ 0.001).

To investigate the underlying mechanisms of isotretinoin-mediated regulation of IL-10 signaling, we studied the expression of proteins along the MAPK p38-MSK1 pathway. Some publications have demonstrated an extra-nuclear function of the retinoic acid receptor (RAR) in fibroblasts, mammary breast cancer cells, keratinocytes and macrophages^26^,^27^. RAR activation by retinoic acid (RA) results in the phosphorylation of MAPK p38-MSK1 proteins that eventually contribute to the expression of IL-10 and DUSP1. Accordingly, we identified an induction of IL-10 signaling (Figs 2 and 4) and an increase of DUSP1 (Supplementary Table 2) in both T-cell subsets of isotretinoin treated animals directly after treatment. When we treated Jurkat cells with isotretinoin we observed only a trend towards an increase of Socs3 protein expression (Fig. 8A) while p38 and MSK1 phosphorylation was not affected (Fig. 8B–D) suggesting that isotretinoin regulates IL-10 and DUSP1 expression through an alternative mechanism in Jurkat cells.

## Discussion

In the present study, we show differential effects of doxycycline, metronidazole and isotretinoin on microRNA and mRNA expression in murine Tregs and naive T-cells with different expression patterns directly after the treatment and at the end of the recovery period. Interestingly, analysis of putative microRNA targets and mRNA expression indicated a direct anti-inflammatory effect for isotretinoin as evidenced by the induction of IL-10 signaling in Tregs and naive T-cells, and a reduction in effector T-cell proliferation indicating no effects that might suggest pathologic effects. Both antibiotics led to a reduced expression of signaling molecules involved in proliferation, which was still observed after the recovery phase in the case of doxycycline. Doxycycline treated animals showed activation of pro-inflammatory pathways in Tregs and naive T-cells directly after the treatment phase, but not after the recovery period. In contrast, metronidazole led to an induction of an anti-inflammatory profile in Tregs immediately after treatment, whereas in naive T-cells this induction was observed only after the recovery period.

The differential microRNA and mRNA expression profile in doxycycline and metronidazole treated animals after the recovery period indicates that these antibiotics have the potential to induce long-term changes in the immune phenotype of Tregs and naive T-cells. These persistent effects on splenic T-cells suggest that both antibiotics have immunosuppressive properties. Isotretinoin induced direct anti-inflammatory effects at microRNA, mRNA and functional level with long-term effects on NFAT-, BCR-, PI3K/AKT-relevant pathways as well was Ca(^2+^)- and G-CSF-mediated processes.The analysis of microRNA expression and their involvement in different cell processes is still challenging due to the lack of normalization strategies and the complexity of microRNA-mRNA interactions. Combined analysis of microRNA and mRNA expression in T-cells applying Next-Generation Sequencing is sparse. Our study shows for the first time a thorough characterization of possible microRNA-mediated mRNA regulation in T-cell subsets that might pave the way for further investigations aimed to understand the contribution of microRNAs to T-cell differentiation and function. Our findings are based on a limited number of biological replicates. For identification of reliably regulated microRNAs we applied strict thresholds in our bioinformatic processing and candidate selection (|log2 (fold change)| ≥1, P-value ≤ 0.001). Yet, the limited number of replicates as well as the strict thresholds for identification of altered microRNA expression might preclude identification of relevant microRNAs. Comparisons between microRNA and mRNA expression are further complicated as sequence based prediction of microRNA targets is limited by the small size of microRNAs and the fact that partial pairing is often sufficient^28^.

Our data set shows, that directly after treatment no correlation between microRNA and mRNA changes can be observed whereas at the later time point some overlap between predicted microRNA targets and differential mRNA expression can be observed. We found no overlap between predicted microRNA targets and differentially expressed mRNAs directly after treatment for none of the applied agents, suggesting that directly after treatment other mechanisms of the complex regulation of gene expression (e.g. chromatin structure and interaction of transcription factors in the regulation of mRNA transcription^29^) outweigh the influence of microRNAs.

So far, only few studies compared the impact of microRNA mediated gene expression regulation with the impact of other mechanisms, but in general the impact of microRNAs is considered weak^30^. In a study investigating the impact of overexpression or knockout of a certain microRNA on protein expression, Baek *et al*. concluded that microRNA probably act to fine-tune protein expression^31^. In some studies a better correlation between microRNA expression and protein levels than between microRNA and mRNA levels was observed^32^. We therefore suggest that directly after the treatment other mechanisms of gene expression regulation superimpose subtle regulations mediated by microRNAs.

One of the goals of our study was to investigate whether isotretinoin or antibiotic treatment can lead to epigenetic modifications that might contribute to the association seen between antibiotic treatment and development of IBD. Epigenetic modifications like DNA methylation and histone modifications can regulate microRNA expression and thereby lead to long-term effects on gene expression regulation^33^. The finding that we do not see an overlap between predicted microRNA targets directly after treatment but after the recovery period may suggest that subtle effects mediated by microRNAS become more visible and effective when stronger, direct mechanisms do not act any more.

We here focused on the comparison of changes at microRNA and mRNA level between isotretinoin, metronidazole and doxycycline treated groups and the respective vehicle control groups directly after the treatment and after the recovery period. In addition, analysis of altered microRNA and mRNA expression within a treatment groups over time provides insight how the genetic signature evolves during the recovery period. However, it is difficult to discriminate between changes over time due to T-cell maturation and changes from treated back to untreated These data can be found in Supplementary Figures 2 and 3 and Supplementary Tables 3 and 4. For identification of differentially expressed microRNAs and mRNAs over time within a treatment group the same thresholds as in the comparison to the control groups (|log2 (fold change)| ≥1, P-value ≤ 0.001 for microRNAs and |log2 (fold change)| ≥0.5, P-value ≤ 0.001 for mRNAs) were applied.

Knowledge on altered microRNA expression in IBD has increased immensely over the past few years. An increase of microRNA-21, -29, -130, -146, -155, -195, -196–214, -223, -375, whereas a decrease of microRNA-10, -124 have been observed in IBD patients^6^,^34^,^35^,^36^,^37^,^38^. Interestingly, in our studies IBD-associated microRNA-130, -195, -196, -223, -375 were down-regulated in Tregs or naive T-cells after isotretinoin treatment, indicating that isotretinoin does not induce a microRNA expression pattern similar to the one observed in IBD patients.

The contribution of retinoic acid (RA) to Treg differentiation is well described^39^,^40^,^41^,^42^. Therefore, it is plausible that isotretinoin, as a RA derivate, might also influence T-cell fate. The role of specific microRNAs in T helper (Th) cell differentiation has been well documented, *e.g.* microRNA-29 in Th1 cells, microRNA-126 in Th2 cells, microRNA-10a in Tregs and microRNA-326 in Th17 cells^43^. Interestingly, microRNA-326 is driving differentiation into Th17 cells by inhibiting Ets-1, a negative regulator of Th17 differentiation. In our study microRNA-326 was down-regulated in naive T-cells after the recovery period, suggesting that differentiation into Th17 cells might be reduced in isotretinoin treated animals.

Interestingly, Socs3, a target gene of RA, was induced immediately after isotretinoin treatment in our *in vivo* and *in vitro* studies. RA in combination with RAR binds to retinoic acid responsive elements (RARE) leading to the expression of RA responsive genes (e.g. *Socs3, Hoxa1, Hoxa4, CRBPs, and Trim16*)^44^. Socs3 has been suggested to have different effects in Teffs and Tregs. In Teffs, Socs3 inhibited STAT4 and STAT3 signaling thereby suppressing Th1 and Th17 responses and favoring Th2 differentiation^45^. In contrast, Tregs overexpression of Socs3 led to decreased proliferation and suppressive function^46^. while T-cell specific loss of Socs3 activated Tregs and resulted in higher production of anti-inflammatory cytokines e.g. IL-10 and TGFβ^47^. Our results showed a significant up-regulation of Socs3 in naive T-cells directly after isotretinoin treatment suggesting a promotion of an anti-inflammatory profile. In Tregs from isotretinoin treated animals we observed a moderate upregulation of Socs3 mRNA along with the induction of mRNAs involved in IL-10 signaling. These findings contrast the observations by Kinjyo and colleagues^46^, indicating the need for further investigations into a possible concentration-dependent role of Socs3 on IL-10 signaling in Tregs. In naive T-cells from isotretinoin treated mice, the expression of Lrrk2, a susceptibility gene for IBD, was increased after the recovery phase. Lrrk2 is an up-stream regulator of NFAT signaling and prevents the latter to translocate into the nucleus, where NFAT has been shown to induce the expression of pro-inflammatory genes^48^,^49^. The overexpression of Lrrk2 in isotretinoin treated animals therefore further supports an anti-inflammatory effect of isotretinoin.

Despite the wide application of antibiotics, their effects on the host immune system (e.g. T-cells) have received limited attention. In particular differential microRNA expression in T-cells after antibiotic treatment has not been thoroughly characterized so far and is shown for the first time in this study.

Recent studies have shown that metronidazole, a nitroimidazole-based antibiotic frequently prescribed for gastrointestinal disorders is strongly associated with new-onset IBD (odds ratio 5.01)^18^,^50^. However, several *in vivo* and *in vitro* studies analyzing metronidazole alone or in combination with other antibiotics (ciprofloxacin, neomycin) showed that metronidazole exhibits direct immunosuppressive effects and ameliorates inflammatory conditions such as colitis^51^,^52^,^53^,^54^,^55^. Importantly, treatment of IL-10^-/-^ mice with neomycin-metronidazole before and after the establishment of colitis ameliorated colitis parameter and diminished adherent bacterial levels indicating protective effects^56^. Accordingly, we also observed an anti-inflammatory effect in splenic Tregs and naive T-cells after treatment with metronidazole. Little is known about the long-term effects mediated by metronidazole in the treatment of IBD. One explanation might be long-term alterations of the gut microbiota although epigenetic modifications in T-cells should also be taken into account.

Tetracyclines have been shown to have immunomodulatory and anti-inflammatory effects in several inflammatory conditions, such as multiples sclerosis, Parkinson’s disease and rheumatoid arthritis^57^,^58^,^59^,^60^. Furthermore, doxycycline suppressed the proliferation of lymphocytes^61^ and minocycline inhibited proliferation and reduced production of IL-2, IFNγ and TNFα in human T-cells^62^. Szeto and colleagues reported that doxycycline inhibited NFAT1 activation in CD4+ T-cells^63^. In contrast, pro-inflammatory effects have been described for doxycycline in murine thymic epithelial cells. Treatment with doxycycline increased the expression of IL-6 and GM-CSF through activation of NFκB and ERK indicating direct pro-inflammatory effects during homeostasis^64^. Additionally, recent epidemiological studies have shown doxycycline to be associated with CD (odds ratio 2.25)^15^. In our study we also observed an induction of pro-inflammatory pathways including NFκB and MAPK signaling in Tregs and naive T-cells directly after treatment with doxycycline. Regarding proliferation, we observed a downregulation of mRNAs involved in cell cycle processes that might indicate decreased proliferation of Tregs. The majority of studies investigated only immediate effects after doxycycline treatment, therefore and to the best of our knowledge, this study is describing for the first time long-term effects of this antibiotic on T-cells. To further elucidate potential long-term effects of isotretionin, doxycycline and metronidazole, analysis of other epigenetic modifications, e.g. changes in chromatin structure by ChIP-Seq or DNA methylation profiling, should be performed. Furthermore, it would be interesting to study epigenetic modifications and functional alterations in human PBMCs in response to isotretinoin, doxycycline and metronidazole treatment to see whether similar effects, as seen here in murine T cell subsets, can be observed.

In summary, microRNA and mRNA expression patterns in naive and regulatory T-cells suggest an anti-inflammatory effect of metronidazole, in contrast to doxycycline, which promoted expression patterns associated with pro-inflammatory processes. Differential expression of microRNAs and mRNAs after the recovery period showed that both antibiotics might induce long-term changes in the immune phenotype in Tregs and naive T-cells. The observed microRNA regulation, directing to immunosuppressive modulation of T-cell function, does not support a strong impact of epigenetic modifications in the association between antibiotic treatment and the development of IBD. Further investigations should address other mechanisms that might mediate long-term effects of drug treatment e.g. changes in gut microbiota composition, to further elucidate causal relationships between drug treatment and later development of IBD.

## Methods

### Mice

Female Balb/c mice were purchased from Charles River Laboratories (Germany) and housed in the animal facility of the University Hospital Zurich under specific pathogen-free conditions and with free access to food and water *ad libitum*.

All animal experiments were approved by the cantonal veterinary office of Zurich under license numbers ZH-54-2011 and ZH-214-2016 and the methods were carried out in accordance with the institutional and state guidelines.

### Design

T-cell samples were collected from each treatment group directly after the treatment period at week 2 (immediate effects), and after a 4-week recovery period (week 6) without any treatment (long-term effects) (Fig. 1).

Animals were randomized according to weight and kept in individual cages according to treatment. The mice were treated daily for 2 weeks by oral gavage with the following doses: isotretinoin 30 ^mg^/_ml_, or rapeseed oil (*Brassica rapa*), metronidazole 107 ^mg^/_kg_, or doxycycline 43 ^mg^/_kg_, or water.

In the experiments with isotretinoin for microRNA expression analysis, we pooled 4 animals to achieve 2 replicates per time point and treatment, whereas for mRNA analysis we used 3–5 replicates due to quantity requirements of total RNA for sequencing. For the antibiotic experiments we pooled 6 animals resulting in 2 replicates per time point and treatment due to quantity requirements of total RNA for sequencing.

### Reagents and antibodies

Isotretinoin was purchased from F. Hoffmann-La Roche Ltd (Basel, Switzerland). Metronidazole, doxycycline and rapeseed oil (*Brassica rapa*) were purchased from Sigma-Aldrich.

In order to investigate the purity of isolated CD4+CD62L+ T-cell fraction, cells were stained with antibodies specific against CD4 (APC-Cy7, GK1.5), CD62L (FITC, MEL-14), CD45RB (Pacific Blue, C363-16A) and CD127 (APC, A7R34). CD4+CD25+ T-cell fraction was stained with antibodies against CD4 (APC-Cy7, GK1.5), CD25 (PE, PC61.5) and Foxp3 (APC, FJK-16s). Antibodies were obtained from either BD Pharmingen (Basel, Switzerland) or eBioscience (San Diego, USA). Data was collected on a FACS Canto II (BD Biosciences, Allschwil, Switzerland) and analysed with FlowJo software (Ashville, USA).

Mouse anti–phospho-MAPK p38 (Thr^180^/Tyr^182^), rabbit anti-p38, rabbit anti–phospho-ERK1/2 (Tyr^42^/Tyr^44^), rabbit anti-ERK1/2 (p44/42 MAPK), rabbit anti-phospho-MSK1 (Thr^581^), rabbit anti-MSK1, rabbit anti-SOCS3 and mouse anti-GAPDH antibodies were obtained from Cell Signaling Technology (Danvers, MA).

### Isolation of murine CD4+CD62L+T-cells and CD4+CD25+ T-cells

CD4+CD62L+ and CD4+CD25+ T-cells were isolated from spleens of female BALB/c mice with the CD4+CD62L+T-cell isolation kit II and CD4+CD25+ regulatory T-cell isolation kit (Miltenyi Biotech, Bergisch Gladbach, Germany) according to the manufacturer’s instructions. In brief, the spleen was homogenised by passing the tissue through a nylon mesh (70 μm) using a plunger. CD4+CD62L+ and CD4+CD25+ T-cells were purified from spleen mononuclear cells in two steps, i.e. depletion of non-CD4+ cells by indirect magnetic labelling with CD4+ T-cell Biotin-Antibody Cocktail II against CD8a, CD11b, CD45R (B220), CD49b, TCRγ/δ and Ter-119 anti-biotin microbeads, followed by further separation of CD4+ T-cells by CD62L (L-selectin) or CD25-PE labelled immunomagnetic beads to positively select CD62L+ and CD25+ T-cells. Cell selection was performed using an AutoMACS separator (MiltenyiBiotec, Germany). The purity of the isolated T-cell populations was checked by FACS analysis (Supplementary Figure 1).

### Treg suppression/Teff proliferation assay and cell culture

After magnetic sorting, responder T-cells (Teff; CD4+CD25-) and regulatory T-cells (Treg; CD4+CD25+) were cultured in a 96-well plate in complete RPMI 1640 medium (Invitrogen, Eugene, OR) which contained 2 mM L-Glutamine (Thermo Fisher, Switzerland) 10% FCS (VWR, USA), 100 ^U^/_ml_ penicillin and 100 ^μg^/_ml_ streptomycin (Sigma-Aldrich), 10 mM HEPES (Thermo Fisher, Switzerland), 0,1 mM nonessential amino acids and 1 mM sodium pyruvate (Sigma-Aldrich, Switzerland). T-cell cultures were maintained at 37 °C, 5% CO_2_ for 4 days. Stimulation of proliferation was achieved with anti-CD3/anti-CD28 coated dynabeads (Thermo Fisher, Switzerland) according to the manufacturer’s instructions.

Responder T-cells (Teff) from vehicle (ctrl) and isotretinoin (iso) treated animals (5 × 10^4^ cells/well) were labeled with 0.5 μM carboxyfluoresceinsuccinimidyl ester (CFSE, Sigma-Aldrich, Switzerland) at 37 °C for 10 min with gentle shaking and washed once with cold complete medium. Subsequently, CFSE-labeled responder T-cells were cultured with Tregs (decreasing numbers of Tregs: 1:2 and 1:8) or unlabeled (unlab) responders as controls. T-cell proliferation was assessed by the decrease of fluorescence of CFSE due to its dilution with each proliferation cycle. Flow cytometry was applied for fluorescence detection.

Human Jurkat cells were cultured in RPMI 1640 medium supplemented with 10% FCS and 1% L-Glutamine. 2 × 10^6^ cells/well were treated with isotretinoin (10 ^ng^/_ml_) for 5 min, 15 min, 1 h, 3 h and 24 h and the mixture of 100 ^ng^/_ml_ PMA, 1 ^mg^/_ml_ CD3, 1 ^mg^/_ml_ CD28 (positive control) at 37 °C, 5% CO_2_. Protein was collected for each time point and analyzed by western blotting.

### Western Blotting

An aliquot of each lysate was mixed with an equal amount of 2 X gel loading buffer (50 ^mmol^/_L_ Tris, pH 6.8, 2% sodium dodecyl sulfate, 200 ^mmol^/_L_ dithiothreitol, 40% glycerol, 0.2% bromophenol blue) and boiled for 5 min. Proteins were separated by sodium dodecyl sulfate polyacrylamide gel electrophoresis and transferred onto nitrocellulose membranes (Thermo Fisher, Switzerland). Membranes were blocked with 5% blocking solution and primary antibody was added in 5% blocking solution overnight. Membranes were washed 3 times with Tris-buffered saline solution containing 1% Tween 20 (1% TBST) for 15 min, horseradish-peroxidase-labelled secondary anti-mouse- or anti-rabbit–IgG-antibody (Santa Cruz Biotechnology) in 5% blocking solution (1:3000) was added for 1 h and membranes were washed 3 times for 15 min with 1% TBST. Finally, immunoreactive proteins were detected using an enhanced chemoluminescence detection kit (GE Healthcare). ImageJ was used for densitometric analysis of the blots.

### Total RNA extraction and quality/quantity analysis

After isolation of CD4+CD62L+ and CD4+CD25+ T-cells with the AutoMACS separator, extraction of total RNA including small RNA was performed with Qiazol (miRNeasy Kit, Qiagen, Germany) according to the manufacturer’s instructions. Before sequencing, total RNA was analysed on the TapeStation2200 (Agilent, Waldbronn, Germany) for defining the quality and quantity of all samples. Samples with a RIN-value > 6 were used for further analysis. The great majority of samples had a RIN-value >9.

### Sequencing procedures

#### mRNA sequencing

##### Library preparation

The TruSeq Stranded mRNA Sample Prep Kit (Illumina, Inc, California, USA) was used as follows: total RNA samples (100 ng) were ribosome depleted and reverse-transcribed into double-stranded cDNA with actinomycin added during first-strand synthesis. The cDNA was fragmented, end-repaired and polyadenylated before ligation of TruSeq adapters. The adapters contain the index for multiplexing. Fragments containing TruSeq adapters on both ends were selectively enriched with PCR. The quality and quantity of the enriched libraries were validated using the Tapestation (Agilent, Waldbronn, Germany). The product is a smear with an average fragment size of approximately 360 bp. The libraries were normalized to 10 nM in Tris-Cl 10 mM, pH 8.5 with 0.1% Tween 20.

##### Cluster Generation and Sequencing

The TruSeq SR Cluster Kit v4-cBot-HS or TruSeq PE Cluster Kit v4-cBot-HS (Illumina, Inc, California, USA) was used for cluster generation using 8 pM of pooled normalized libraries on the cBOT. Sequencing was performed on the Illumina HiSeq 2500 single read at 1 × 125 bp using the TruSeq SBS Kit v4-HS (Illumina, Inc, California, USA).

#### microRNA sequencing

##### Library preparation

The NEB Next Multiplex Small RNA Sample Prep Kit (New England Biolabs, Inc, MA, USA) was used in the following steps. Briefly, 3’- and 5′-RNA adapters were ligated to total RNA samples (1 μg) in 2 steps. Ligated samples were reverse-transcribed into double-stranded cDNA. Fragments containing TruSeq adapters on both ends were selectively enriched with PCR. The 145-160 bp small RNA fractions were selected on 6% polyacrylamide gels. The quality and quantity of the enriched libraries were validated using the Tapestation (Agilent, Waldbronn, Germany). The product is a smear with an average fragment size of approximately 150 bp. The libraries were normalized to 10 nM in Tris-Cl 10 mM, pH 8.5 with 0.1% Tween 20.

##### Cluster Generation and Sequencing

The TruSeq PE Cluster Kit v3-cBot-HS (Illumina, Inc, California, USA) was used for cluster generation using 10 pM of pooled normalized libraries on the cBOT. Sequencing was performed on the Illumina HiSeq 2000 Single end at 1 × 50 bp using the TruSeq SBS Kit v3-HS (Illumina, Inc, California, USA). Data Analysis of small RNA-seq reads were quality-checked with fastqc which computes various quality metrics for the raw reads.

#### Bioinformatics

##### Data pre-processing:

The raw fastqc reads were first cleaned by removing adapter sequences, trimming low quality ends, and filtering reads with low quality (phred quality <20) using Trimmomatic^65^. In case of small RNA reads only the adapter sequences were removed.

##### mRNA-Sequencing

Sequence alignment of the resulting high-quality reads to the *Mus musculus* reference genome (build GRCm38) and quantification of gene level expression was carried out using RSEM (Version 1.2.21)^66^. To detect differentially expressed genes, we applied the count based negative binomial model implemented in the software package edgeR (R version: 3.2.0, edgeR version: 3.10.2)^67^, in which the normalization factor was calculated by trimmed mean of M values (TMM) method^68^. The gene-wise dispersions were estimated by conditional maximum likelihood and an empirical Bayes procedure was used to shrink the dispersions towards a consensus value. The differential expression was assessed using an exact test adapted for over-dispersed data. Genes showing altered expression with adjusted P-value ≤ 0.05 were considered differentially expressed (Benjamini and Hochberg method). Further selection of mRNAs was done with the following threshold: |log2 (fold change)| ≥0.5, P-value ≤ 0.001.

##### microRNA-Sequencing

Sequence alignment, quantification and quality control were performed using ncPRO (version 1.5.1)^69^. The microRNA annotation is based on miRBase version 20. Differentially expressed microRNAs were identified applying edgeR. Further selection of microRNAs was done with the following thresholds: |log2 (fold change)| ≥1, P-value ≤ 0.001.

##### microRNA targets and mRNA correlation

Predicted microRNA targets were analyzed with TargetScan (Version 6.2) and TargetScan custom (Version 5.2). Pathway analyses were performed with MetaCore by Thomson Reuters (Version 6.24). For identification of enriched pathways the Metacore variation of the Fisher’s exact test and correction for multiple sample testing by FDR was applied.

### Statistical Analysis

Statistical comparisons were performed by two tailed Student’s test. P-values ≤ 0.05 were considered statistically significant.

## Additional Information

**How to cite this article**: Becker, E. *et al*. Doxycycline, metronidazole and isotretinoin: Do they modify microRNA/mRNA expression profiles and function in murine T-cells? *Sci. Rep.* **6**, 37082; doi: 10.1038/srep37082 (2016).

**Publisher’s note**: Springer Nature remains neutral with regard to jurisdictional claims in published maps and institutional affiliations.

## Supplementary Material

Supplementary Information

Supplementary Information

Supplementary Information

Supplementary Information

Supplementary Information
