# Supplementary material for: Doxycycline, metronidazole and isotretinoin: Do they modify microRNA/mRNA expression profiles and function in murine T-cells?
Source: Sci Rep. 2016 Nov 17;6:37082. doi: 10.1038/srep37082 (PMC5113073; doi:10.1038/srep37082)

Doxycycline, metronidazole and isotretinoin: Do they modify microRNA/mRNA expression profiles and function in murine T-cells?

Eugenia Becker[1], *Susan Bengs*[1], *Sirisha Aluri*[2], *Lennart Opitz*[2], *Kirstin Atrott*[1], *Claudia Stanzel*[1], *Pedro A. Ruiz Castro*[1], *Gerhard Rogler*[1] and *Isabelle Frey-Wagner*[1*]

[1]Division of Gastroenterology and Hepatology, University Hospital Zurich, Zurich, Switzerland

[2]Functional Genomics Center Zurich, Zurich, Switzerland

*Address for correspondence:

Dr. Isabelle Frey-Wagner
Division of Gastroenterology and Hepatology
University Hospital Zurich,
Raemistr. 100
8091 Zurich, Switzerland
Phone: +41-44-2559916
Fax: +41-44-2559497
E-Mail: Isabelle.Frey@usz.ch

Supplementary figure 1:

Post sorting purity of T-cells fractions. FACS analysis of effector T-cells (Teff, CD3+CD4+CD25-), regulatory T-cells (Treg, CD4+CD25+ Foxp3+) and naive T-cells (CD4+CD62L+CD45RB+CD127+). Total numbers and representative graphs are shown for each treatment and time point. No differences in cell numbers were observed in the different groups or time points.

Supplementary figure 2:

Full-length blots of in vitro mechanistic studies in Jurkat cells. Jurkat cells were treated with isotretinoin (10 $^{ng}/_{ml}$) and PMA (100 $^{ng}/_{ml}$) in combination with anti-CD3/CD28 stimulation and samples were taken at the indicated time points. Representative full-length blots show levels of Socs3, GAPDH, p-ERK (Tyr^{42}/Tyr^{44}) and total ERK, p-p38 (Thr^{180}/Tyr^{182}) and total p38 and p-MSK1 (Thr^{581}) and total MSK1 with molecular size markers. Antibodies were used according to the manufacturer's instructions. Processing of brightness and contrast were applied on all blots equally.

Supplementary figure 3:

Differentially expressed microRNAs (A) and mRNAs (B) in T-cells within the studied groups over time.

Supplementary table 1:

Differentially expressed microRNAs in T-cells in response to isotretinoin, metronidazole or doxycycline treatment directly after treatment (immediate effect) or after a 4-week recovery period (long-term effect). Differentially expressed microRNAs in response to the individual treatments and time points in Tregs and naive T-cells as generated with Next-Generation Sequencing (Illumina HighSeq 2500). The microRNA annotation is based on miRBase version 20. The detection of differentially expressed microRNAs was done using *edgeR*.

MicroRNAs were considered as significantly different compared to the control group with the following threshold: |log2 (fold change)| ≥ 1, P-value ≤ 0.001.

<u>Supplementary table 2:</u>

Differentially expressed mRNAs in T-cells in response to isotretinoin, metronidazole or doxycycline treatment directly after treatment (immediate effect) or after a 4-week recovery period (long-term effect). Differential expression of mRNAs after the three treatment courses and time points in naive T and Tregs as generated with Next-Generation Sequencing (Illumina HighSeq 2500). mRNAs were considered as significantly different compared to the control group with the following threshold: |log2 (fold change)| ≥ 0.5, P-value ≤ 0.001.

<u>Supplementary table 3:</u>

Differentially expressed microRNAs in T-cells within the studied groups over time. Differentially expressed microRNAs in Tregs and naive T-cells within the studied groups over time as generated with Next-Generation Sequencing (Illumina HighSeq 2500). The microRNA annotation is based on miRBase version 20. The detection of differentially expressed microRNAs was done using edgeR. MicroRNAs were considered as significantly different compared to the control group with the following threshold: |log2 (fold change)| ≥ 1, P-value ≤ 0.001.

<u>Supplementary table 4:</u>

Differentially expressed mRNAs in T-cells within the studied groups over time. Differential expression of mRNAs in naive T and Tregs within the studie groups over time as generated with Next-Generation Sequencing (Illumina HighSeq 2500). mRNAs were considered as significantly different compared to the control group with the following threshold: |log2 (fold change)| ≥ 0.5, P-value ≤ 0.001.

Supplementary Figure 1

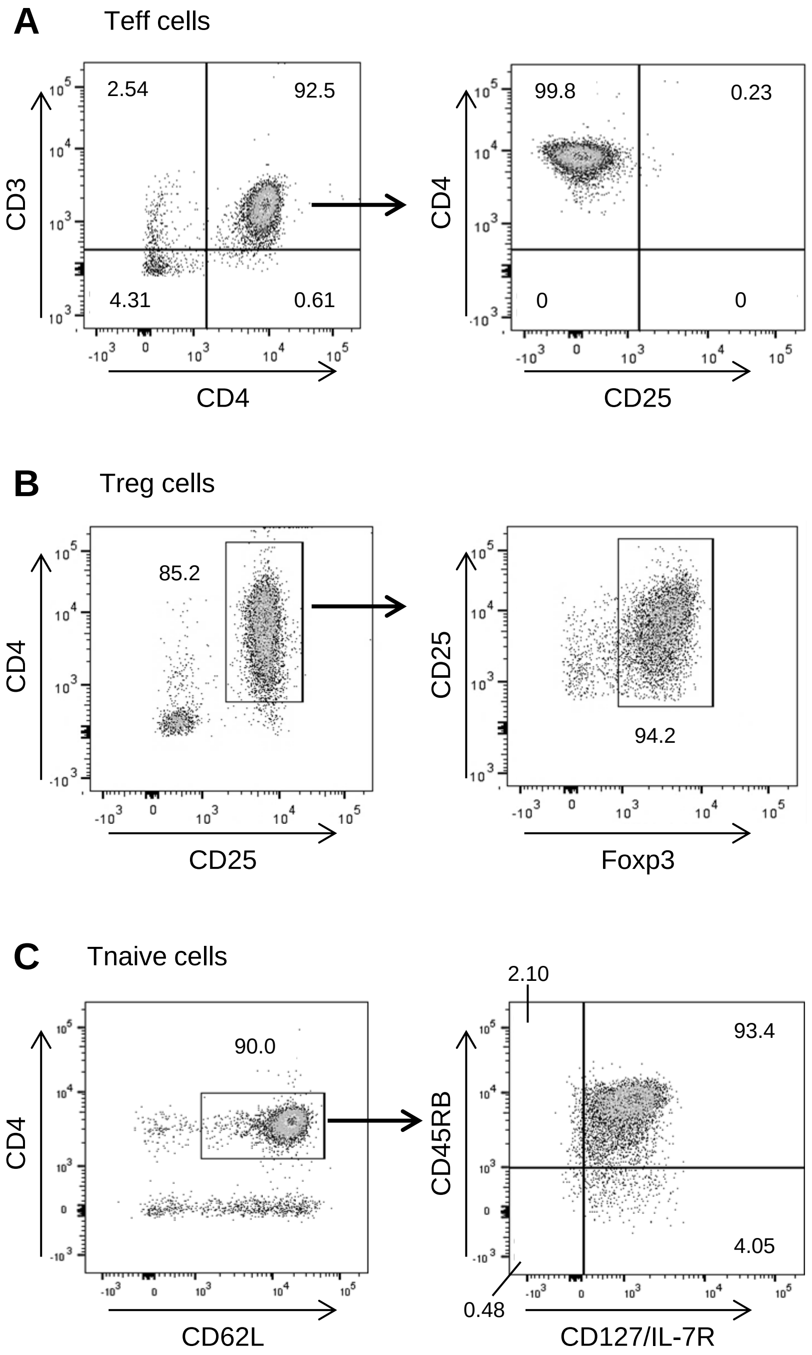

Supplementary Figure 2

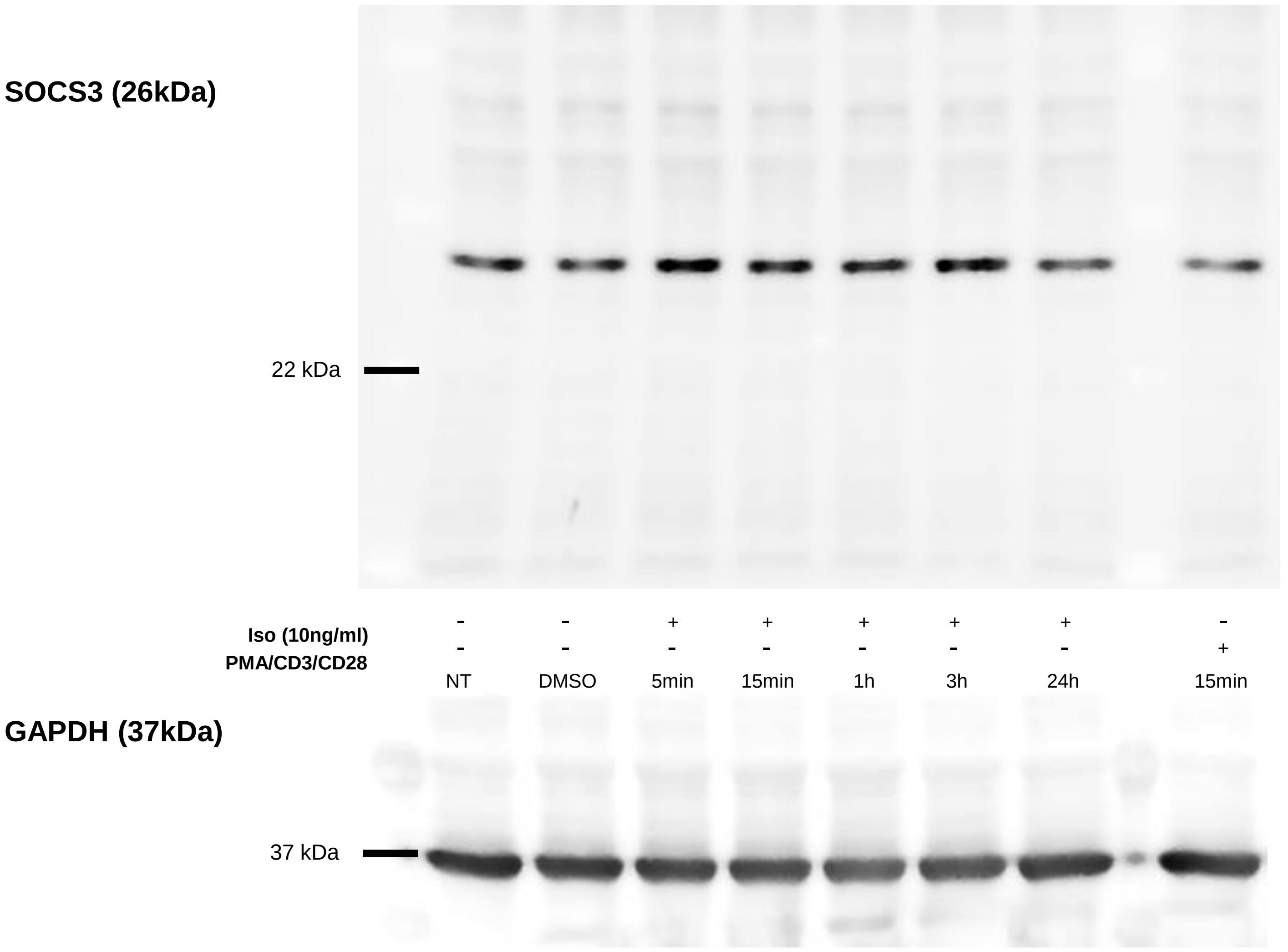

P-ERK1/2 (44kDa)

Total ERK1/2 (44kDa)

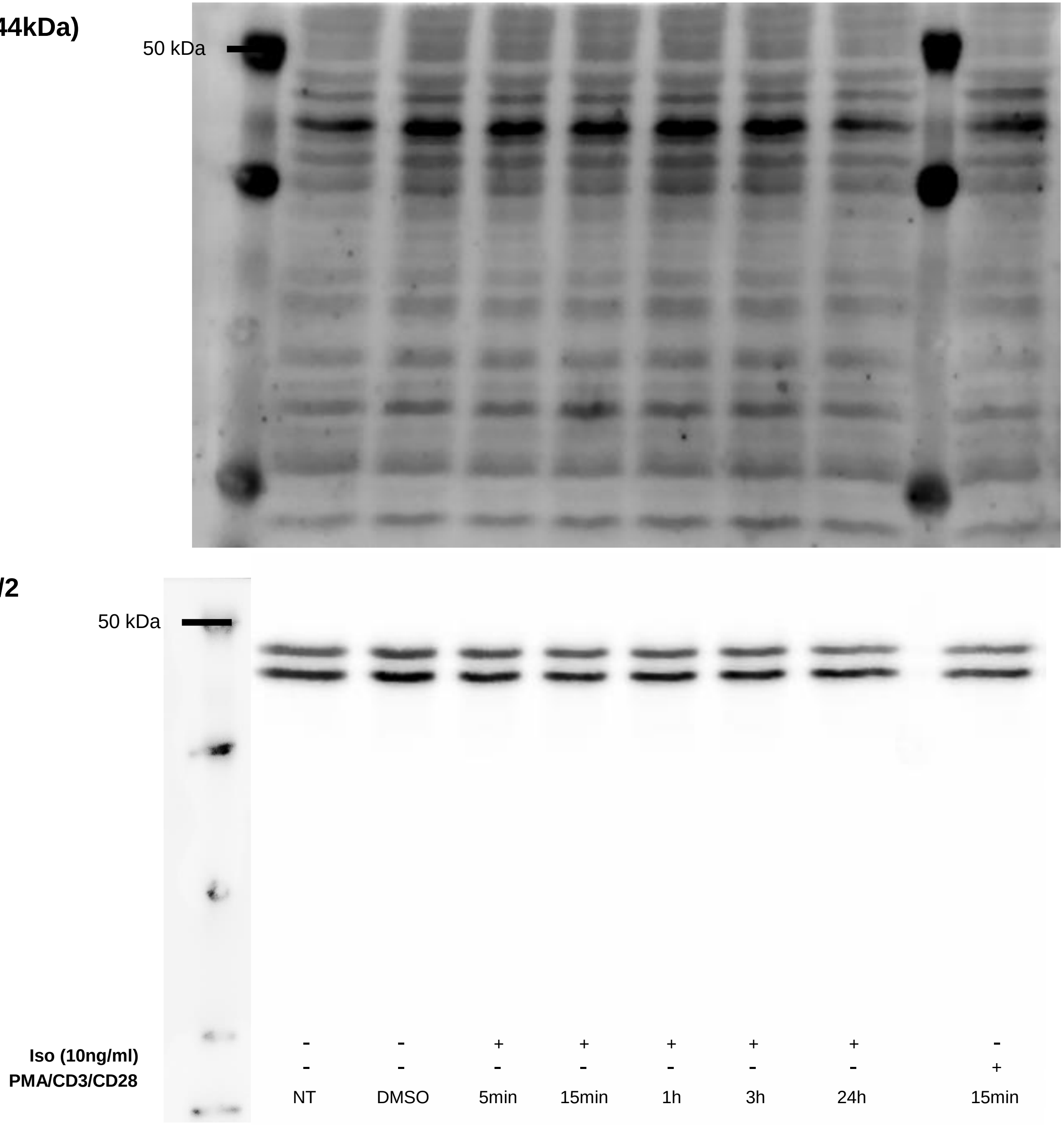

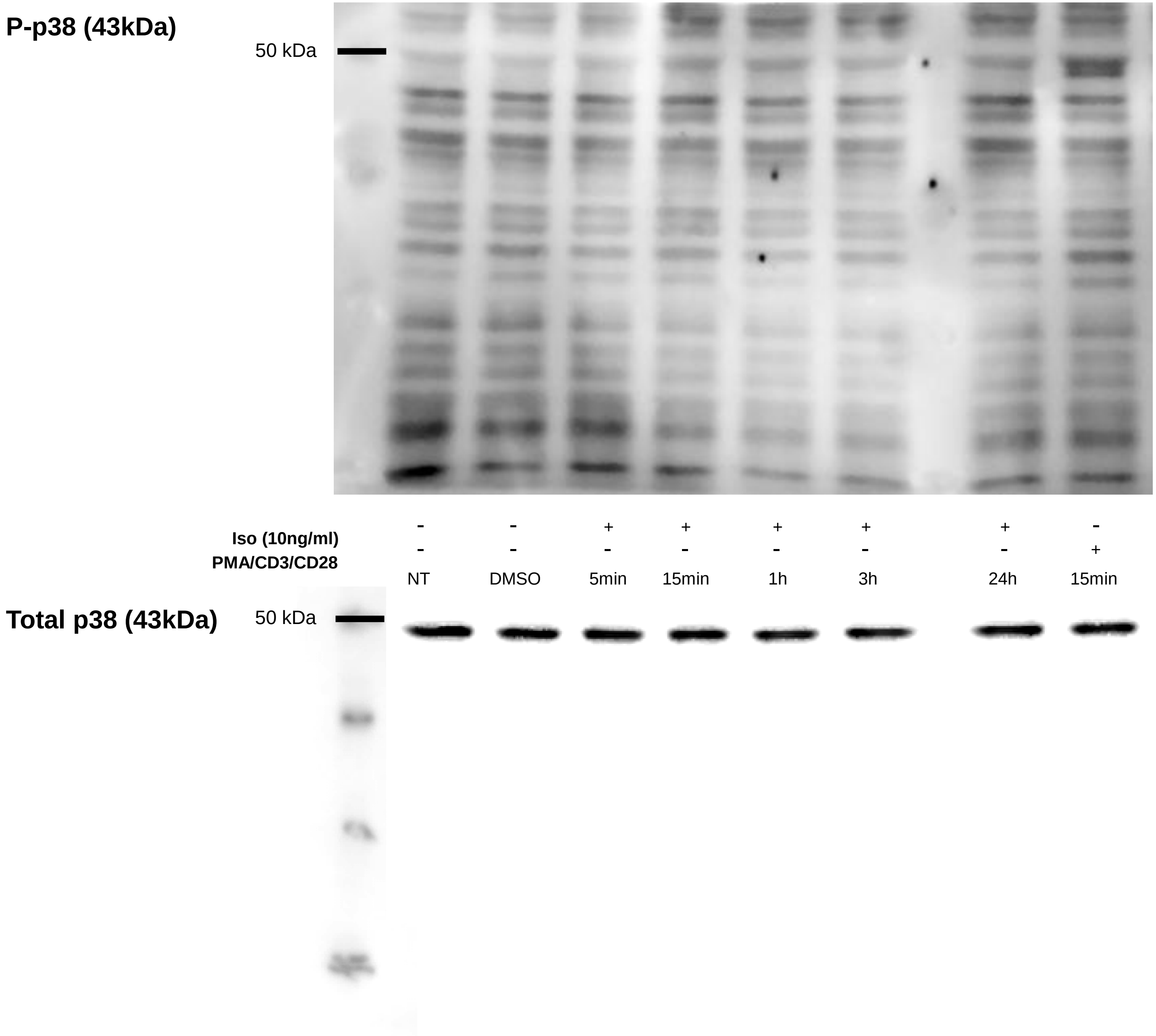
P-p38 (43kDa)
50 kDa
Iso (10ng/ml) - - + + + + + -
PMA/CD3/CD28 - - - - - - - +
NT DMSO 5min 15min 1h 3h 24h 15min
Total p38 (43kDa)
50 kDa

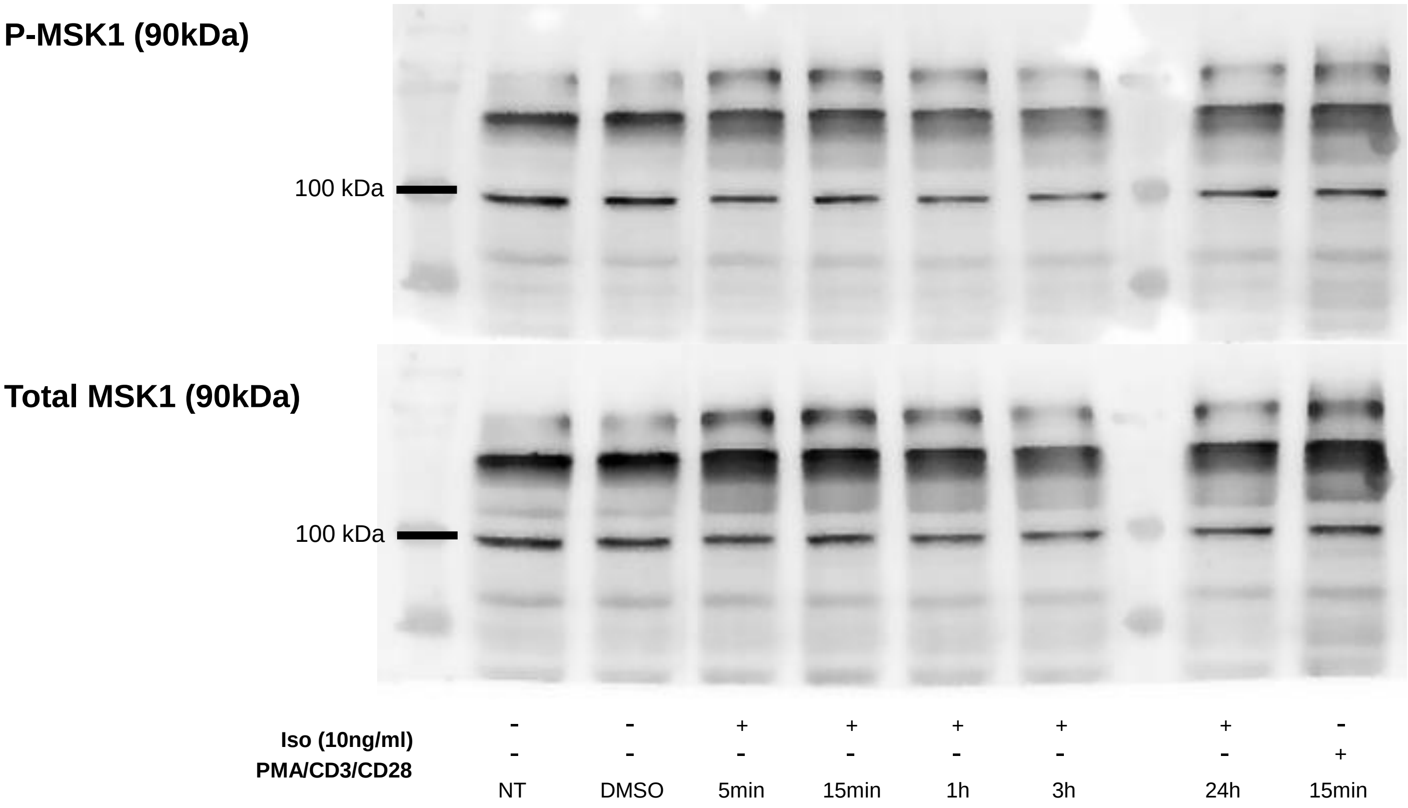
P-MSK1 (90kDa)
100 kDa
Total MSK1 (90kDa)
100 kDa
Iso (10ng/ml)
PMA/CD3/CD28
- - + + + + + -
- - - - - - - +
NT DMSO 5min 15min 1h 3h 24h 15min

Supplementary Figure 3 A

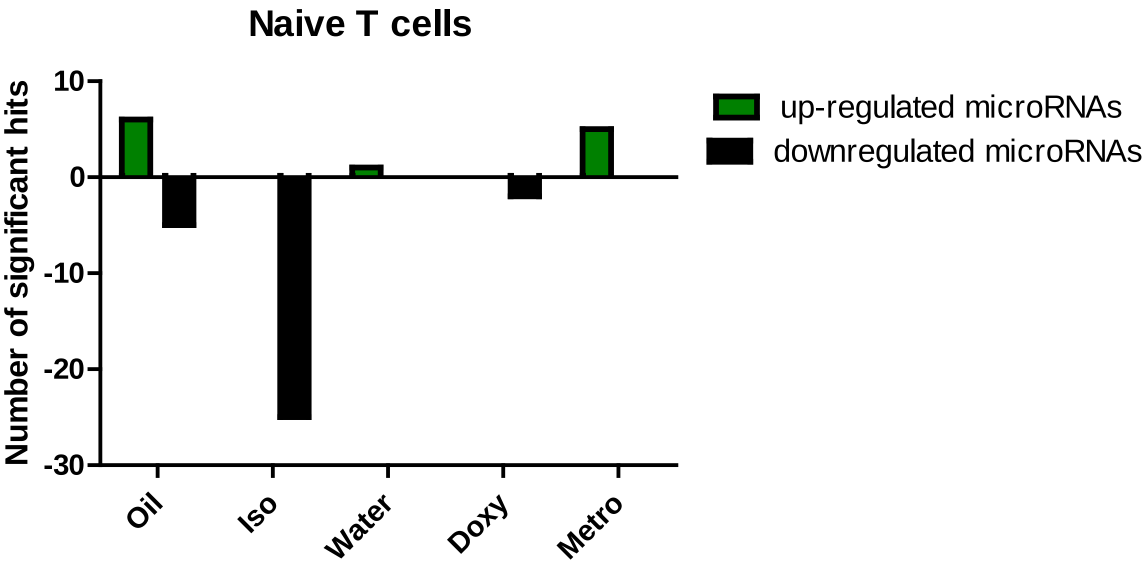

Differentially expressed microRNAs within a group over time

expression after the recovery period vs. expression directly after treatment

thresholds for identification of differentially expressed microRNAs:

|log2 (fold change)| ≥ 1,

P-value ≤ 0.001

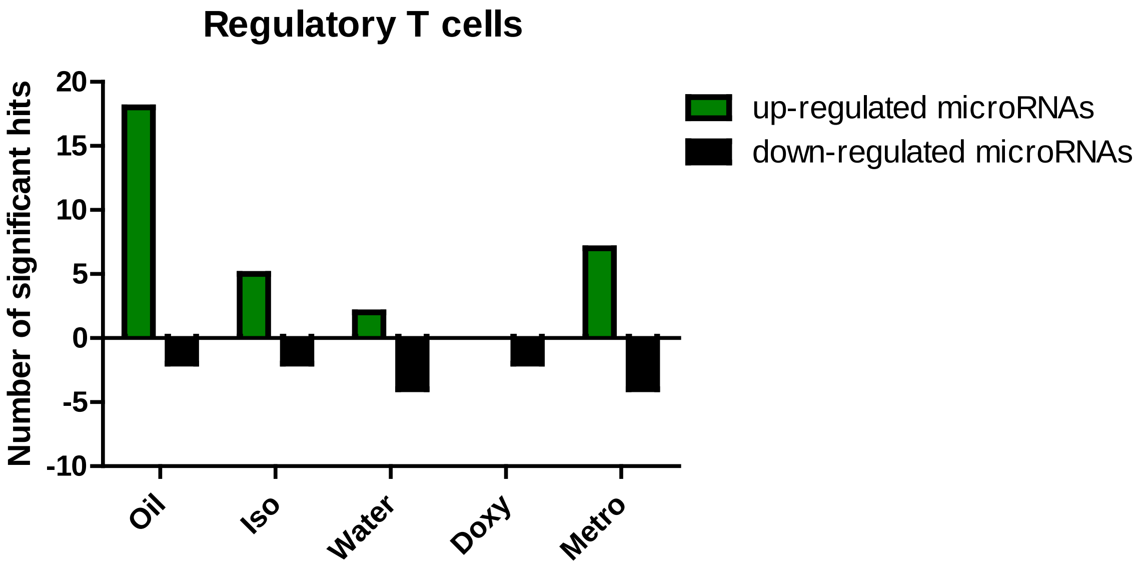

Supplementary Figure 3 B

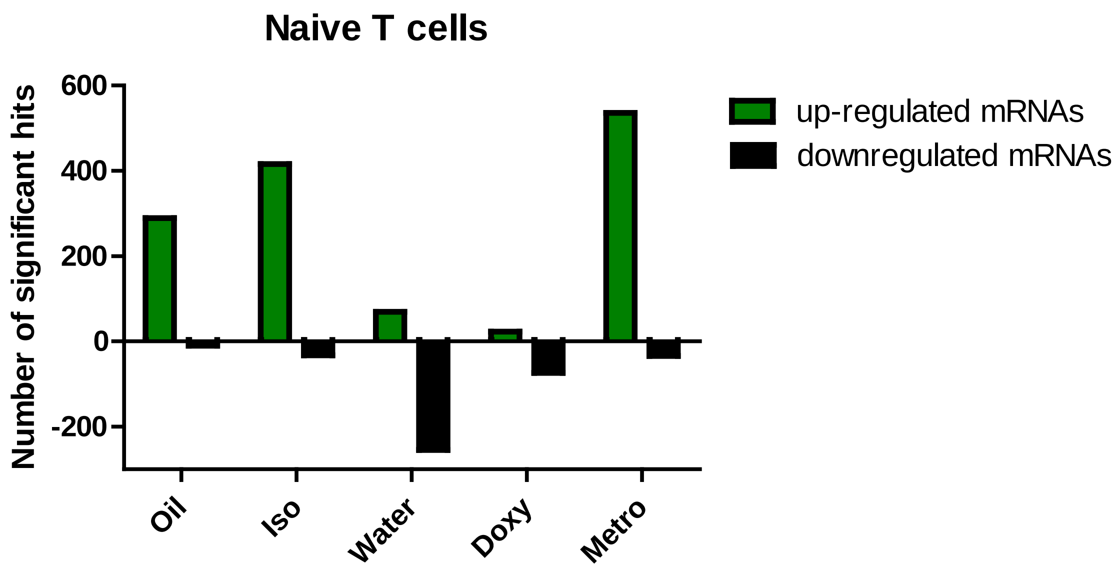

Differentially expressed mRNAs within a group over time

expression after the recovery period vs. expression directly after treatment

thresholds for identification of differentially expressed microRNAs:

|log2 (fold change)| ≥ 0.05,

P-value ≤ 0.001

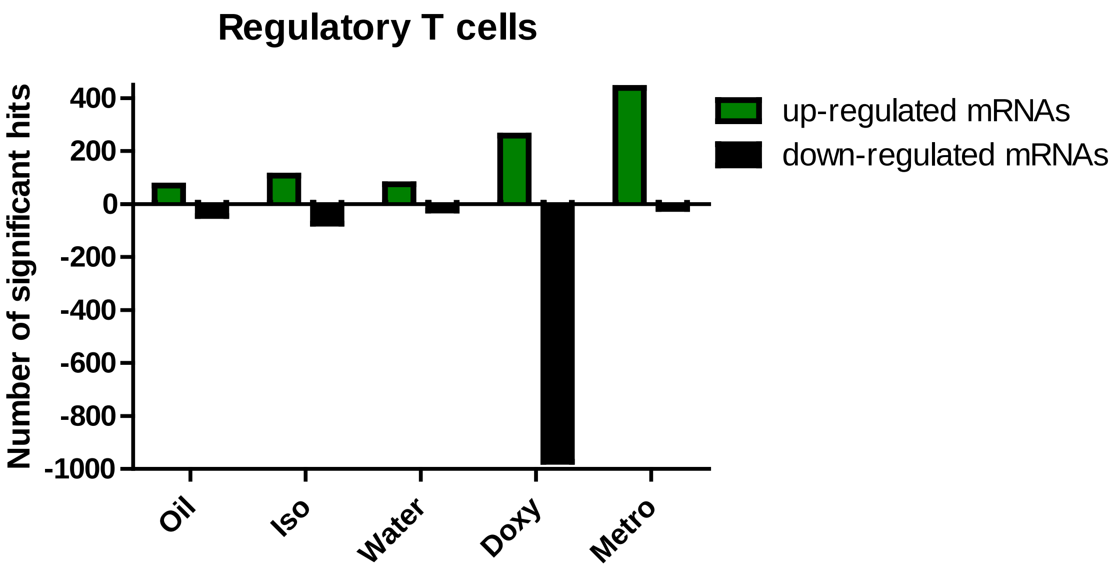

Supplement: Supplementary Information [file srep37082-s1.pdf]
